# 2-(4-Methyl­benzene­sulfonamido)-2-phenyl­acetic acid

**DOI:** 10.1107/S1600536809042299

**Published:** 2009-10-17

**Authors:** Irfana Mariam, Mehmet Akkurt, Shahzad Sharif, Noreen Akhtar, Islam Ullah Khan

**Affiliations:** a Materials Chemistry Laboratory, Department of Chemistry, Government College University, Lahore 54000, Pakistan; b Department of Physics, Faculty of Arts and Sciences, Erciyes University, 38039 Kayseri, Turkey

## Abstract

In the title compound, C_15_H_15_NO_4_S, the dihedral angle between the phenyl and benzene rings is 46.0 (3)° and a weak intra­molecular N—H⋯O inter­action is present. The crystal structure is stabilized by inter­molecular O—H⋯O, N—H⋯O and C—H⋯O hydrogen bonds.

## Related literature

For previous studies on the synthesis of sulfonamide derivatives with phenyl glycine, see: Asiri *et al.* (2009 ▶); Arshad *et al.* (2009 ▶). For reference structural data, see: Allen *et al.* (1987 ▶).
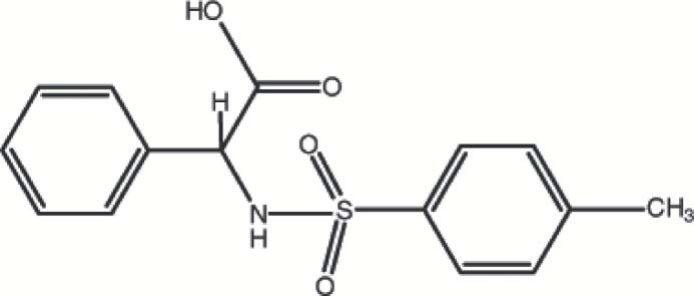


## Experimental

### 

#### Crystal data

C_15_H_15_NO_4_S
*M* *_r_* = 305.35
Orthorhombic, 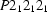

*a* = 5.6592 (12) Å
*b* = 11.208 (2) Å
*c* = 23.342 (4) Å
*V* = 1480.5 (5) Å^3^
*Z* = 4
Mo *K*α radiation
μ = 0.23 mm^−1^
*T* = 296 K
0.35 × 0.22 × 0.10 mm

#### Data collection

Bruker Kappa APEXII CCD diffractometer
Absorption correction: refined from Δ*F* (*XABS2*; Parkin *et al.*, 1995 ▶) *T*_min_ = 0.923, *T*_max_ = 0.977
3753 measured reflections
3753 independent reflections
1502 reflections with *I* > 2σ(*I*)

#### Refinement

*R*[*F* ^2^ > 2σ(*F* ^2^)] = 0.066
*wR*(*F* ^2^) = 0.192
*S* = 0.94
3753 reflections
195 parameters
H atoms treated by a mixture of independent and constrained refinement
Δρ_max_ = 0.30 e Å^−3^
Δρ_min_ = −0.38 e Å^−3^
Absolute structure: Flack (1983 ▶), 1550 Freidel pairs
Flack parameter: −0.11 (19)

### 

Data collection: *APEX2* (Bruker, 2007 ▶); cell refinement: *SAINT* (Bruker, 2007 ▶); data reduction: *SAINT*; program(s) used to solve structure: *SHELXS97* (Sheldrick, 2008 ▶); program(s) used to refine structure: *SHELXL97* (Sheldrick, 2008 ▶); molecular graphics: *ORTEP-3 for Windows* (Farrugia, 1997 ▶); software used to prepare material for publication: *WinGX* (Farrugia, 1999 ▶) and *PLATON* (Spek, 2009 ▶).

## Supplementary Material

Crystal structure: contains datablocks global, I. DOI: 10.1107/S1600536809042299/hb5142sup1.cif

Structure factors: contains datablocks I. DOI: 10.1107/S1600536809042299/hb5142Isup2.hkl

Additional supplementary materials:  crystallographic information; 3D view; checkCIF report

## Figures and Tables

**Table 1:** Hydrogen-bond geometry (Å, °)

*D*—H⋯*A*	*D*—H	H⋯*A*	*D*⋯*A*	*D*—H⋯*A*
O1—H*O*1⋯O2^i^	0.82	1.85	2.655 (6)	168
N1—H*N*1⋯O1^ii^	0.85 (5)	2.47 (5)	3.251 (6)	154 (5)
N1—H*N*1⋯O2	0.85 (5)	2.43 (5)	2.748 (6)	103 (4)
C7—H7⋯O3^iii^	0.98	2.43	3.343 (7)	155

Symmetry codes: (i) 
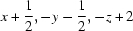
; (ii) 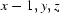; (iii) 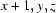.

**Fig. 1.**
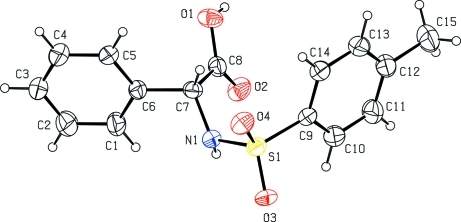
An ORTEP-3 view of the title molecule with the atom numbering scheme. Displacement ellipsoids for non-H atoms are drawn at the 30% probability level.

**Fig. 2.**
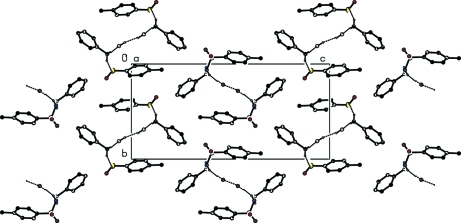
The packing and hydrogen bonding of the title compound viewed down a axis. Hydrogen atoms not involved in the showed interactions have been omitted for clarity.

## supplementary crystallographic information

### Comment

(*R*)-alpha-Amino-benzeneacetic is a side chain component of Ampicillin, Cephalexin and Cephaclor. Cephalexin has *D*-phenylglycyl group as a substituent at the 7-amino position and an unsubstituted methyl group at the 3-position. This is in connection with our previous study on synthesis of sulfonamide derivatives with phenyl glycine (Arshad *et al.*, 2009).

In the title molecule (I), (Fig. 1), bond lengths (Allen *et al.*, 1987) and bond angles are in the range of expected values. The planes of the phenyl and benzene rings (C1–C6) and (C9–C14) make a dihedral angle of 46.0 (3) ° with each other.

In the structure, the adjacent molecules are connected by intermolecular O—H···O, N—H···O and C—H···O hydrogen bonds (Table 1). In Fig. 2, the packing and hydrogen bonding of (I) are shown viewed down *a* axis.

### Experimental

Phenyl glycine (1.0 g, 6.6 mmol) was dissolved in 20 ml distilled in a round bottom flask (100 ml). 1*M* Na_2_CO_3_ solution was used to maintain pH at 8–9. Para-toluene sulfonyl chloride (1.26 g, 6.6 mmol) was added to the solution, and stirred at room temperature until the para-toluene sulfonylchloride was consumed. On completion of the reaction, while vigorous stirring pH was adjusted 1–2, using 1 *M* HCl. The precipitate formed in this way was filtered off, washed with distilled water, dried and recrystalized in methanol and ethyl acetate (50:50 *v*/*v*) to yield light brown prisms of (I).

### Refinement

The NH H atom was localized from the difference-Fourier map and its coordinates were refined freely. The isotropic temperature parameters of the H atom were calculated as 1.2*U*_eq_ of the parent atom. H atoms were located geometrically and treated as riding with C—H = 0.98 Å (methine), C—H = 0.96 Å (methyl), C—H = 0.93 Å (aromatic) and O—H = 0.82 Å (hydroxyl) with *U*_iso_(H) = 1.2 or 1.5*U*_eq_(C, O).

### Crystal data

**Table:**

C_15_H_15_NO_4_S	*F*(000) = 640
*M**_r_* = 305.35	*D*_x_ = 1.370 Mg m^−^^3^
Orthorhombic, *P*2_1_2_1_2_1_	Mo *K*α radiation, λ = 0.71073 Å
Hall symbol: P 2ac 2ab	Cell parameters from 533 reflections
*a* = 5.6592 (12) Å	θ = 2.5–15.0°
*b* = 11.208 (2) Å	µ = 0.23 mm^−^^1^
*c* = 23.342 (4) Å	*T* = 296 K
*V* = 1480.5 (5) Å^3^	Prism, light brown
*Z* = 4	0.35 × 0.22 × 0.10 mm

### Data collection

**Table:**

Bruker Kappa APEXII CCD diffractometer	3753 independent reflections
Radiation source: sealed tube	1502 reflections with *I* > 2σ(*I*)
graphite	*R*_int_ = 0.0000
φ and ω scans	θ_max_ = 28.6°, θ_min_ = 1.7°
Absorption correction: part of the refinement model (Δ*F*) (*XABS2*; Parkin *et al.*, 1995)	*h* = −7→7
*T*_min_ = 0.923, *T*_max_ = 0.977	*k* = 0→15
3753 measured reflections	*l* = 0→31

### Refinement

**Table:**

Refinement on *F*^2^	Secondary atom site location: difference Fourier map
Least-squares matrix: full	Hydrogen site location: inferred from neighbouring sites
*R*[*F*^2^ > 2σ(*F*^2^)] = 0.066	H atoms treated by a mixture of independent and constrained refinement
*wR*(*F*^2^) = 0.192	*w* = 1/[σ^2^(*F*_o_^2^) + (0.0657*P*)^2^] where *P* = (*F*_o_^2^ + 2*F*_c_^2^)/3
*S* = 0.94	(Δ/σ)_max_ < 0.001
3753 reflections	Δρ_max_ = 0.30 e Å^−^^3^
195 parameters	Δρ_min_ = −0.38 e Å^−^^3^
0 restraints	Absolute structure: Flack (1983), 1550 Freidel pairs
Primary atom site location: structure-invariant direct methods	Flack parameter: −0.11 (19)

### Special details

**Table:**

Experimental. Absorption correction: XABS2; Parkin *et al.* (1995), linear fit to sin(theta)/lambda - 12 parameters
Geometry. Bond distances, angles *etc*. have been calculated using the rounded fractional coordinates. All su's are estimated from the variances of the (full) variance-covariance matrix. The cell e.s.d.'s are taken into account in the estimation of distances, angles and torsion angles
Refinement. Refinement on *F*^2^ for ALL reflections except those flagged by the user for potential systematic errors. Weighted *R*-factors *wR* and all goodnesses of fit *S* are based on *F*^2^, conventional *R*-factors *R* are based on *F*, with *F* set to zero for negative *F*^2^. The observed criterion of *F*^2^ > σ(*F*^2^) is used only for calculating -*R*-factor-obs *etc*. and is not relevant to the choice of reflections for refinement. *R*-factors based on *F*^2^ are statistically about twice as large as those based on *F*, and *R*-factors based on ALL data will be even larger.

### Fractional atomic coordinates and isotropic or equivalent isotropic displacement parameters (Å^2^)

**Table:**

	*x*	*y*	*z*	*U*_iso_*/*U*_eq_	
S1	0.0144 (3)	0.06971 (13)	0.90449 (6)	0.0506 (5)	
O1	0.6089 (8)	−0.1884 (4)	0.93717 (17)	0.0620 (16)	
O2	0.2309 (8)	−0.2174 (4)	0.96274 (18)	0.0683 (17)	
O3	−0.2356 (6)	0.0816 (4)	0.90143 (16)	0.0590 (16)	
O4	0.1628 (7)	0.1504 (4)	0.87441 (17)	0.0607 (17)	
N1	0.0702 (7)	−0.0631 (4)	0.87997 (19)	0.0470 (17)	
C1	0.2019 (12)	−0.2702 (6)	0.8097 (3)	0.070 (3)	
C2	0.2455 (14)	−0.3397 (7)	0.7628 (3)	0.085 (3)	
C3	0.4491 (12)	−0.3245 (6)	0.7318 (3)	0.073 (3)	
C4	0.6069 (13)	−0.2379 (6)	0.7467 (3)	0.072 (3)	
C5	0.5634 (9)	−0.1673 (6)	0.7937 (3)	0.061 (3)	
C6	0.3604 (10)	−0.1822 (5)	0.8253 (2)	0.0463 (19)	
C7	0.3143 (9)	−0.1052 (5)	0.8779 (2)	0.0470 (19)	
C8	0.3763 (11)	−0.1745 (5)	0.9306 (2)	0.050 (2)	
C9	0.0950 (9)	0.0722 (5)	0.9766 (2)	0.0490 (19)	
C10	−0.0428 (11)	0.0200 (5)	1.0177 (3)	0.062 (2)	
C11	0.0257 (12)	0.0163 (6)	1.0734 (3)	0.070 (3)	
C12	0.2384 (12)	0.0650 (6)	1.0907 (3)	0.067 (2)	
C13	0.3774 (11)	0.1171 (6)	1.0498 (3)	0.065 (3)	
C14	0.3091 (10)	0.1222 (5)	0.9926 (3)	0.057 (2)	
C15	0.3157 (14)	0.0600 (8)	1.1524 (3)	0.099 (3)	
H1	0.06550	−0.28220	0.83110	0.0840*	
HO1	0.63490	−0.22620	0.96660	0.0930*	
H2	0.13660	−0.39750	0.75180	0.1010*	
HN1	−0.020 (9)	−0.116 (5)	0.894 (2)	0.0560*	
H3	0.47970	−0.37340	0.70040	0.0880*	
H4	0.74340	−0.22670	0.72520	0.0870*	
H5	0.67200	−0.10900	0.80420	0.0730*	
H7	0.41830	−0.03540	0.87600	0.0560*	
H10	−0.18660	−0.01380	1.00720	0.0740*	
H11	−0.07210	−0.01950	1.10040	0.0840*	
H13	0.52160	0.15010	1.06050	0.0780*	
H14	0.40570	0.15860	0.96550	0.0690*	
H15A	0.48070	0.04040	1.15420	0.1490*	
H15B	0.22600	0.00020	1.17220	0.1490*	
H15C	0.28970	0.13620	1.17010	0.1490*	

### Atomic displacement parameters (Å^2^)

**Table:**

	*U*^11^	*U*^22^	*U*^33^	*U*^12^	*U*^13^	*U*^23^
S1	0.0449 (8)	0.0563 (8)	0.0505 (8)	0.0024 (8)	0.0026 (8)	0.0009 (8)
O1	0.051 (2)	0.074 (3)	0.061 (3)	−0.001 (2)	−0.012 (2)	0.021 (2)
O2	0.066 (3)	0.082 (3)	0.057 (3)	0.003 (2)	0.016 (2)	0.017 (2)
O3	0.034 (2)	0.076 (3)	0.067 (3)	0.0123 (19)	−0.0037 (19)	−0.002 (2)
O4	0.059 (3)	0.055 (3)	0.068 (3)	−0.002 (2)	0.013 (2)	0.012 (2)
N1	0.040 (3)	0.054 (3)	0.047 (3)	0.003 (2)	0.001 (2)	−0.002 (2)
C1	0.070 (4)	0.078 (5)	0.062 (4)	−0.014 (4)	0.008 (4)	−0.024 (4)
C2	0.082 (5)	0.083 (6)	0.089 (5)	−0.015 (4)	0.001 (4)	−0.034 (4)
C3	0.065 (5)	0.095 (6)	0.060 (4)	0.016 (4)	0.001 (3)	−0.022 (4)
C4	0.062 (4)	0.096 (6)	0.059 (5)	0.007 (4)	0.011 (4)	−0.009 (4)
C5	0.044 (4)	0.085 (5)	0.053 (4)	0.002 (3)	0.003 (3)	−0.009 (3)
C6	0.048 (3)	0.052 (4)	0.039 (3)	0.003 (3)	0.000 (3)	0.000 (3)
C7	0.038 (3)	0.057 (4)	0.046 (3)	−0.004 (2)	−0.002 (2)	0.001 (3)
C8	0.053 (4)	0.057 (4)	0.040 (3)	−0.008 (3)	0.002 (3)	−0.003 (3)
C9	0.038 (3)	0.056 (3)	0.053 (4)	0.004 (3)	0.004 (3)	−0.007 (3)
C10	0.055 (4)	0.069 (4)	0.062 (4)	−0.008 (3)	0.004 (3)	−0.005 (3)
C11	0.075 (5)	0.084 (5)	0.052 (4)	−0.003 (4)	0.005 (4)	0.000 (3)
C12	0.071 (4)	0.073 (4)	0.056 (4)	0.010 (4)	−0.006 (4)	−0.010 (4)
C13	0.053 (4)	0.069 (4)	0.072 (5)	−0.003 (3)	−0.004 (4)	−0.014 (4)
C14	0.050 (4)	0.062 (4)	0.060 (4)	0.000 (3)	0.003 (3)	−0.007 (3)
C15	0.108 (6)	0.126 (7)	0.064 (5)	0.017 (6)	−0.017 (4)	−0.019 (5)

### Geometric parameters (Å, °)

**Table:**

S1—O3	1.423 (4)	C10—C11	1.357 (10)
S1—O4	1.420 (4)	C11—C12	1.382 (10)
S1—N1	1.626 (5)	C12—C13	1.368 (10)
S1—C9	1.744 (5)	C12—C15	1.506 (10)
O1—C8	1.334 (8)	C13—C14	1.391 (10)
O2—C8	1.213 (7)	C1—H1	0.9300
O1—HO1	0.8200	C2—H2	0.9300
N1—C7	1.461 (7)	C3—H3	0.9300
N1—HN1	0.85 (5)	C4—H4	0.9300
C1—C6	1.382 (9)	C5—H5	0.9300
C1—C2	1.366 (10)	C7—H7	0.9800
C2—C3	1.371 (10)	C10—H10	0.9300
C3—C4	1.364 (10)	C11—H11	0.9300
C4—C5	1.375 (10)	C13—H13	0.9300
C5—C6	1.375 (8)	C14—H14	0.9300
C6—C7	1.523 (7)	C15—H15A	0.9600
C7—C8	1.497 (7)	C15—H15B	0.9600
C9—C14	1.386 (8)	C15—H15C	0.9600
C9—C10	1.368 (8)		
			
O3—S1—O4	120.2 (3)	C13—C12—C15	121.1 (6)
O3—S1—N1	105.1 (2)	C12—C13—C14	121.8 (6)
O3—S1—C9	107.9 (2)	C9—C14—C13	119.0 (6)
O4—S1—N1	107.1 (2)	C2—C1—H1	120.00
O4—S1—C9	108.2 (3)	C6—C1—H1	120.00
N1—S1—C9	107.7 (3)	C1—C2—H2	120.00
C8—O1—HO1	109.00	C3—C2—H2	120.00
S1—N1—C7	119.4 (3)	C2—C3—H3	120.00
S1—N1—HN1	113 (4)	C4—C3—H3	120.00
C7—N1—HN1	111 (4)	C3—C4—H4	120.00
C2—C1—C6	120.1 (6)	C5—C4—H4	120.00
C1—C2—C3	120.3 (7)	C4—C5—H5	120.00
C2—C3—C4	120.3 (7)	C6—C5—H5	120.00
C3—C4—C5	119.7 (6)	N1—C7—H7	108.00
C4—C5—C6	120.5 (6)	C6—C7—H7	108.00
C1—C6—C5	119.2 (5)	C8—C7—H7	108.00
C1—C6—C7	120.4 (5)	C9—C10—H10	119.00
C5—C6—C7	120.4 (5)	C11—C10—H10	119.00
N1—C7—C6	111.8 (4)	C10—C11—H11	120.00
C6—C7—C8	109.2 (4)	C12—C11—H11	119.00
N1—C7—C8	111.2 (4)	C12—C13—H13	119.00
O2—C8—C7	123.7 (5)	C14—C13—H13	119.00
O1—C8—C7	112.7 (5)	C9—C14—H14	121.00
O1—C8—O2	123.5 (5)	C13—C14—H14	120.00
S1—C9—C10	121.4 (4)	C12—C15—H15A	109.00
S1—C9—C14	119.7 (4)	C12—C15—H15B	109.00
C10—C9—C14	118.8 (5)	C12—C15—H15C	110.00
C9—C10—C11	121.5 (6)	H15A—C15—H15B	109.00
C10—C11—C12	121.1 (6)	H15A—C15—H15C	109.00
C11—C12—C15	121.2 (6)	H15B—C15—H15C	109.00
C11—C12—C13	117.8 (6)		
			
O3—S1—N1—C7	−179.2 (4)	C1—C6—C7—N1	−45.4 (7)
O4—S1—N1—C7	51.8 (4)	C1—C6—C7—C8	78.1 (7)
C9—S1—N1—C7	−64.3 (4)	C5—C6—C7—N1	136.5 (5)
O3—S1—C9—C10	36.7 (6)	C5—C6—C7—C8	−100.0 (6)
O3—S1—C9—C14	−146.9 (5)	N1—C7—C8—O1	−164.1 (4)
O4—S1—C9—C10	168.2 (5)	N1—C7—C8—O2	18.5 (7)
O4—S1—C9—C14	−15.4 (5)	C6—C7—C8—O1	72.0 (6)
N1—S1—C9—C10	−76.3 (5)	C6—C7—C8—O2	−105.3 (6)
N1—S1—C9—C14	100.1 (5)	S1—C9—C10—C11	176.5 (5)
S1—N1—C7—C6	−143.9 (4)	C14—C9—C10—C11	0.0 (9)
S1—N1—C7—C8	93.8 (5)	S1—C9—C14—C13	−176.2 (5)
C6—C1—C2—C3	1.6 (11)	C10—C9—C14—C13	0.4 (8)
C2—C1—C6—C5	−1.2 (10)	C9—C10—C11—C12	−0.2 (10)
C2—C1—C6—C7	−179.4 (6)	C10—C11—C12—C13	0.0 (10)
C1—C2—C3—C4	−1.6 (11)	C10—C11—C12—C15	−179.4 (7)
C2—C3—C4—C5	1.3 (11)	C11—C12—C13—C14	0.3 (10)
C3—C4—C5—C6	−0.9 (10)	C15—C12—C13—C14	179.8 (6)
C4—C5—C6—C1	0.9 (9)	C12—C13—C14—C9	−0.5 (9)
C4—C5—C6—C7	179.1 (6)		

### Hydrogen-bond geometry (Å, °)

**Table:**

*D*—H···*A*	*D*—H	H···*A*	*D*···*A*	*D*—H···*A*
O1—HO1···O2^i^	0.82	1.85	2.655 (6)	168
N1—HN1···O1^ii^	0.85 (5)	2.47 (5)	3.251 (6)	154 (5)
N1—HN1···O2	0.85 (5)	2.43 (5)	2.748 (6)	103 (4)
C7—H7···O3^iii^	0.98	2.43	3.343 (7)	155

Symmetry codes: (i) *x*+1/2, −*y*−1/2, −*z*+2; (ii) *x*−1, *y*, *z*; (iii) *x*+1, *y*, *z*.

## References

[bb1] Allen, F. H., Kennard, O., Watson, D. G., Brammer, L., Orpen, A. G. & Taylor, R. (1987). *J. Chem. Soc. Perkin Trans. 2*, pp. S1–19.

[bb2] Arshad, M. N., Tahir, M. N., Khan, I. U., Shafiq, M. & Ahmad, S. (2009). *Acta Cryst.* E**65**, o940. 10.1107/S1600536809011611 PMC2977641 21583984

[bb3] Asiri, A. M., Akkurt, M., Khan, S. A., Arshad, M. N., Khan, I. U. & Sharif, H. M. A. (2009). *Acta Cryst.* E**65**, o1246–o1247. 10.1107/S1600536809016900 PMC2969753 21583112

[bb4] Bruker (2007). *APEX2* and *SAINT* Bruker AXS Inc., Madison, Wisconsin, USA.

[bb5] Farrugia, L. J. (1997). *J. Appl. Cryst.* **30**, 565.

[bb6] Farrugia, L. J. (1999). *J. Appl. Cryst.* **32**, 837–838.

[bb7] Flack, H. D. (1983). *Acta Cryst.* A**39**, 876–881.

[bb8] Parkin, S., Moezzi, B. & Hope, H. (1995). *J. Appl. Cryst.* **28**, 53–56.

[bb9] Sheldrick, G. M. (2008). *Acta Cryst.* A**64**, 112–122. 10.1107/S0108767307043930 18156677

[bb10] Spek, A. L. (2009). *Acta Cryst.* D**65**, 148–155. 10.1107/S090744490804362X PMC2631630 19171970

